# An Investigation of the Influence of the Sequence of Laser Texturing and Heat Treatment Processes on the Coefficient of Friction of X165CrMoV12 Steel

**DOI:** 10.3390/ma19091781

**Published:** 2026-04-28

**Authors:** Yavor Sofronov, Boyan Dochev, Antonio Nikolov, Krum Petrov, Valentin Mishev, Rayna Dimitrova, Milko Yordanov, Milko Angelov, Georgi Todorov, Krassimir Marchev

**Affiliations:** 1 Department of Theory of Mechanisms and Machines, Faculty of Industrial Technology, Technical University of Sofia, 1756 Sofia, Bulgaria; 2 Department of Mechanics, Faculty of Mechanical Engineering, Technical University of Sofia—Branch Plovdiv, 4000 Plovdiv, Bulgaria; 3 Department of Material Science and Technology of Materials, Faculty of Industrial Technology, Technical University of Sofia, 1756 Sofia, Bulgaria; anikolov@tu-sofia.bg (A.N.); kpetrov@tu-sofia.bg (K.P.); v_mishev@tu-sofia.bg (V.M.); r_dimitrova@tu-sofia.bg (R.D.); 4 Department of Mechanical Engineering, Manufacturing Engineering and Thermal Engineering, Faculty of Engineering and Pedagogy—Branch Sliven, Technical University of Sofia, 8800 Sliven, Bulgaria; m_yordanov@tu-sofia.bg; 5 Faculty of Industrial Technology, Technical University of Sofia, 1756 Sofia, Bulgaria; milko.angelov@tu-sofia.bg (M.A.); k.marchev@northeastern.edu (K.M.); 6 Center of Excellence “Mechatronics and Clean Technology”—Campus Studentski Grad, Technical University of Sofia, 1756 Sofia, Bulgaria; gdt@tu-sofia.bg; 7 Department of Manufacturing Technology and Systems, Faculty of Industrial Technology, Technical University of Sofia, 1756 Sofia, Bulgaria; 8 College of Professional Studies, Northeastern University, Boston, MA 02115, USA

**Keywords:** laser modification, heat treatment, coefficient of friction

## Abstract

The effect of nanosecond laser modification on X165CrMoV12 tool steel before and after heat treatment was investigated. Three laser texturing modes were applied to the studied material, with the variables being the frequency used and the pulse energy: 50 kHz/pulse energy 0.9 mJ, 100 kHz/pulse energy 0.45 mJ, and 150 kHz/pulse energy 0.3 mJ. The other parameters of laser texturing were power—90%; speed—500 mm/s; hatching angle—0° (horizontal), +60°/−60° (or equivalent 120°), and +30°/−30° (or equivalent 150°); and Hatching Distance—0.02 mm. The surface laser modification process aims to obtain a homogeneous and adaptive surface relief optimizing the operational properties of the working surfaces of the parts under dry contact friction conditions. The influence of the used laser modification modes on the roughness class of the obtained surfaces, the structure of the formed modified surface and the friction coefficient was studied. The comparative analysis showed that the lowest roughness class (Ra—4.123 µm) was obtained when using an operating frequency of 50 kHz. The obtained friction coefficient values were lowest in the following sequence of processes: laser texturing and subsequent thermal treatment. The lowest friction coefficient (µ = 0.0041) was registered in the test bodies processed with a mode in which the operating frequency was 50 kHz and the pulse energy was 0.9 mJ, after which they were subjected to thermal treatment according to the used cycle. In this processing sequence, no diffusion-related defects (decarburization) were observed on the surface layer of the tested steel.

## 1. Introduction

Laser modification (texturing) of the surface of steel parts has been established as a precise method that has been experimentally validated and allows for targeted modification of the tribological, mechanical and physicochemical properties of the surface layer, without affecting the structure and characteristics of the material in its volume. This method provides the possibility of contactless, local and reproducible processing, in which micro- and nanostructures with a precisely defined geometry are created. In this way, it differs from more conventional methods of mechanical or chemical processing. Thus, laser texturing is not just a technological process, but a tool for controlling the functional characteristics of the surface of products [1]. The main effect achieved by laser texturing is mainly related to the possibility of controlling the actual contact area between the rubbing surfaces and changing the local distribution of contact stresses. By creating micro-indentations, channels or lattice patterns, favorable conditions are provided for the retention and distribution of the lubricant film, as well as for trapping wear particles outside the direct contact zone. This in turn stabilizes the tribological behavior and leads to a decrease in both the friction coefficient and the wear intensity [2]. It should not be overlooked that the morphology and effectiveness of the textures depend largely on the parameters of the laser process—power, scanning speed, number of cycles and processing strategy. They determine the depth, shape and regularity of the resulting structures [3,4,5].

X165CrMoV12 steel is a high-carbon, high-chromium (≈12 wt% Cr) martensitic tool steel characterized by excellent wear resistance, high hardness (60–62 HRC) and good toughness. Increasing the hardness (60–62 HRC) and improving the performance properties of the steel are achieved by heat treatment (quenching and tempering). Usually tempering is carried out two or three times after quenching, the purpose of which is to reduce the amount of residual austenite and form and separate fine carbides (MC and M2C). Based on the fact that chromium is the main alloying element, chromium carbides of the Cr7C and Cr23C6 types are preferentially formed. This material has found application in the manufacture of tools for cold plastic deformation: punching and drawing dies, precision dies, gauges, cutting tools (machine knives for cutting material up to 6 mm thick), thread rolling tools and other parts subject to wear in the process of work. This steel is also used for the manufacture of injection molds for the production of polymer products, as well as for the manufacture of tools for the production of aluminum alloy parts by pressure casting (operating temperatures lower than 300 °C).

As mentioned, laser modification of the surface of steel parts is a validated precise method for modifying the tribological, mechanical and physicochemical properties of the surface layer by forming micro- and nanostructures with a precisely defined geometry. Although laser texturing does not affect the structure and characteristics of the material in its volume, in steels alloyed with elements with different boiling points (evaporation), their selective evaporation in the treated areas is possible. In X165CrMoV12 steel, the element chromium has the lowest boiling point (2671 °C) compared to vanadium (3407 °C) and molybdenum (4639 °C), which implies a loss of Cr in the treated areas during laser texturing. In cases of laser ablation in the absence of a protective inert gas, the carbon in the surface layer reacts extremely intensively with atmospheric oxygen and burns in the form of CO and CO_2_. When performing laser texturing of X165CrMoV12 steel in the absence of inert gas, due to the high temperatures of the process, there is a possibility of the loss of carbon and chromium in the surface layer, which in turn will lead to a change in its chemical composition, structure and properties.

Currently, much research is focused on studying the possibility of applying hard coatings to previously laser-textured surfaces. The combination of laser texturing with diamond-like carbon (DLC) coatings is an actively researched area in the field of surface engineering [6,7,8]. Femtosecond and nanosecond laser microstructuring deserve special attention among laser technologies, mainly due to their extremely short pulse duration. This feature minimizes the thermal impact on the material. Studies in the field of diamond-like nanocomposite coatings have shown that femtosecond texturing improves the tribological behavior in lubricating friction. Detailed analyses of the microstructuring reveal possibilities for very precise control over the shape and distribution of individual geometric microelements [9,10,11,12,13,14,15,16]. Along with DLC types of coatings, nitrides such as CrN and TiN have also been the subject of extensive research [17,18,19]. Complex nitride compositions such as AlCrSiN are also not far behind in this respect. Studies with laser-textured AlCrSiN surfaces have shown increased adhesion and frictional stability [20,21,22,23,24,25]. Optimization of laser process parameters has shown a clear relationship with wear resistance and overall machining efficiency [26].

For tool steels, available studies show that laser texturing alone can improve tribological properties, even without the application of an additional coating, thereby reducing friction and wear [27]. However, for stainless steels, the combination of laser texturing with a metallic coating provides even better functional surface improvement [28]. Periodic nanostructures on hard coatings, such as TiN, demonstrate a clear relationship between morphology and tribological properties [29]. There is a confirmed synergistic effect when laser texturing is combined with DLC coatings on steel [30,31]. Ongoing studies on AlCrSiN, AlSiTiN and CrN coatings highlight that the microgeometry of the texture is essential for wear resistance [32,33,34,35]. Review publications systematize the main mechanisms and applications of laser texturing in DLC coatings [36]. Studies conducted in some sources have shown that the orientation of the texture relative to the direction of friction significantly affects the tribological behavior, and similar patterns have been found in compressor coatings. Laser-textured stainless steels, for example, show extended lubricant film retention and reduced wear under boundary lubrication conditions [37,38,39]. Adhesion of coatings depends on the shape and size of the texture elements. Systematic studies have shown that optimized texture improves mechanical bonding and reduces residual stress, and the shape of the texture plays a key role [39,40,41,42,43,44,45].

Recent studies on tool and stainless steels, as well as on composite structures with textured coatings, confirm the real industrial potential of laser texturing as a technology for optimizing the surface characteristics of materials [28,46], including 3D-printed iron polylactic acid (Ir-PLA) [47].

However, in real production conditions it is not always possible to apply hard coatings to textured surfaces, which in turn requires improving the operational properties of the parts by using conventional methods of heat treatment. Based on the presented data, the purpose of the present study is also formulated.

The aim of the present work is to investigate the influence of the sequence of the processes of laser texturing and heat treatment on the tribological behavior of X165CrMoV12 steel and to obtain data on the influence of the used operating frequency during texturing—50 kHz, 100 kHz, or 150 kHz—on the roughness class and the friction coefficient of the laser-modified surfaces.

## 2. Materials and Methods

The research presented in this work is related to the tool steel X165CrMoV12 BDS EN ISO 4957:2018 (Tool steels (ISO 4957:2018) European Standard, Bulgarian Institute for Standardization, Sofia, Bulgaria, 17 December 2018 [48]), which is first laser-textured and then subjected to heat treatment (quenching and tempering), as well as the reverse sequence of processes: first conducting heat treatment and subsequent laser modification. The used tool X165CrMoV12 steel was delivered in the form of a bar material in an annealed state with a hardness of 250 HB from which test specimens with dimensions of diameter Ø = 32 mm and thickness = 5 mm were made. The chemical composition of the steel according to the relevant standard [48] is shown in Table 1.

**Figure 1:**
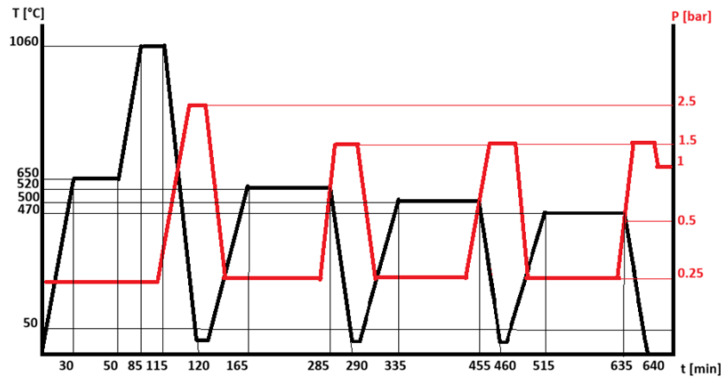
Thermal cycle for quenching and triple tempering of X165CrMoV12 steel (the black line shows the operating temperature and the red line the pressure of the gas used).

Tool steel X165CrMoV12 is a high-carbon, highly alloyed martensitic steel characterized by high wear resistance, good impact toughness and high compressive strength. The increase in hardness (60–62 HRC) and the improvement in the operational properties of the steel are achieved by heat treatment (quenching and tempering). The problem in carrying out the quenching process is related to the high carbon content in the steel. The recommended heating temperatures before quenching are 1020–1060 °C, at which larger amounts of carbon dissolve in the austenite, which in turn leads to a larger amount of residual austenite in the martensite structure after quenching. This in turn is the reason for carrying out double or triple tempering after quenching, the purpose of which is to reduce the amount of residual austenite and form and separate fine carbides (MC and M_2_C). Because chromium is a major alloying element, chromium carbides of the Cr_7_C and Cr_23_C_6_ types are preferentially formed. It is known that when heating steels for hardening in facilities where a protective environment is not used, decarburization of the surface layer is observed—an undesirable defect, which is strongly expressed in steels with a high carbon content (X165CrMoV12). The loss of carbon in the surface layers leads to a change in their chemical composition, which in turn is the reason for not obtaining the desired structures and, thus, properties in these areas. This necessitates the use of a protective environment during the heating process for hardening. Phase changes occur during the heating process for hardening, which is characterized by volume changes and in turn requires the correct choice of the heating rate in the different temperature ranges, as well as of the amount of time the material is kept there in order to fully complete the conversion and dissolution processes. Based on the presented data, the thermal cycle for quenching and tempering of the studied X165CrMoV12 steel was also selected. The thermal treatment included heating to a temperature of 650 °C for 30 min, holding at this temperature for 20 min, and subsequently heating to the selected working temperature of 1060 °C for homogenization of the structure. The heating time from 650 °C to 1060 °C was 35 min, and the holding time at this temperature was 30 min. The heating took place in a nitrogen (N_2_) environment with a pressure of 0.25 bar, and the quenching process was carried out in a nitrogen environment, but with an increased pressure—2.5 bar. For the complete transformation of austenite into martensite after quenching, three-fold tempering was applied. The first annealing after quenching involved heating to a temperature of 520 °C (for 45 min) and holding at this temperature for 120 min; the process was carried out again in a nitrogen environment with a pressure of 0.25 bar, and during the cooling process the nitrogen pressure was increased to 1.5 bar. In the second annealing cycle, the operating temperature was 500 °C, and in the third cycle it was 470 °C, with the heating and holding times not changing, nor the nitrogen control parameters (Figure 1).

**Figure 2:**
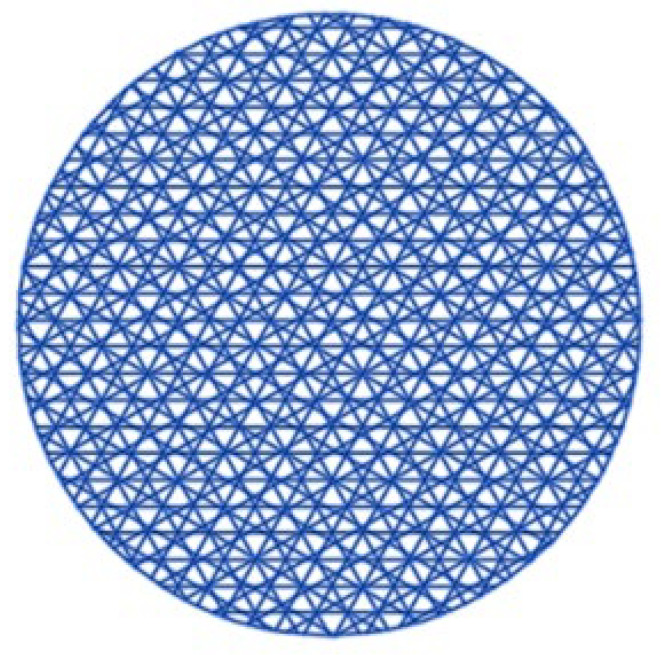
Texturing strategy—multi-scale texture.

The texturing strategy was implemented with a nanosecond fiber optic laser “GF400U” at three different laser modes, with the only variable being the frequency, as follows: Mode 1—50 kHz/pulse energy 0.9 mJ, Mode 2—100 kHz/pulse energy 0.45 mJ, and Mode 3—150 kHz/pulse energy 0.3 mJ. Other parameters of laser texturing were power—90%; speed—500 mm/s; frequency—50–150 kHz; hatching angle—0° (horizontal), +60°/−60° (or equivalent 120°), and +30°/−30° (or equivalent 150°); and Hatching Distance—0.02 mm. The selected modes (power 90%, speed 500 mm/s, frequency 50–150 kHz, pulse 150 ns, and hatch 0.02 mm) were tailored to create a uniform functional microtexture without causing thermal damage to the substrate. The selected power (90%) provides sufficient energy for processing hard carbides, compensates for reflectivity and maintains high process performance. A speed of 500 mm/s reduces thermal heating and limits the thermal influence zone, thus ensuring a stable texturing process, and the chosen operating frequency (50–150 kHz) allows for precise dosing of the pulse energy and the nature of the texture. This texturing strategy (Figure 2) was chosen because it provides isotropic, uniform and scalable surface modification without dominant orientation.

To investigate the influence of the sequence of laser texturing and heat treatment processes on the coefficient of friction (µ) of X165CrMoV12 steel, tribological tests were conducted using a tribotester from Jinan Hengxu Test Technology Co., Ltd., Jinan, China. The test was conducted under dry friction conditions at room temperature, the normal load was 10N, the friction speed (V) was 100 rpm, and the friction time was 3 min (friction path 24 m). The method used was ball-on-disk with three spheres with a diameter d = 6.37 mm, located at an angle of 120° to each other, the spheres being located at a diameter d = 25 mm relative to the axis of the rotary head and made of 100Cr6 steel (hardness 830 HV0.1).

Before conducting the tribological tests, the roughness of the test bodies was measured using a portable contact profilometer–roughness meter from INSIZE ISR—C002, China (Insize, Suzhou City, China). Before conducting the measurements, the device was calibrated with a control sample of roughness Ra = 1.2 µm, and before each measurement, a correction of the position of the device relative to the measured sample was carried out [49,50].

## 3. Results

To study the influence of the sequence of laser texturing and heat treatment processes on the friction coefficient of X165CrMoV12 steel, the following experiments were conducted: quenching and tempering of test specimens from the studied steel and subsequent laser texturing, as well as laser ablation of the test specimens and subsequent heat treatment. Before conducting the tribological studies, the roughness of the test specimens was measured after the three texturing modes with different operating frequencies: 50 kHz, 100 kHz, and 150 kHz (Table 2).

Based on the fact that the roughness of the test bodies before laser texturing was Ra 0.607 µm, the laser texturing process leads to a significant change in the roughness of the treated surfaces. The results obtained show that the lowest roughness is measured for the test bodies where the operating frequency used was 50 kHz. With increasing texturing frequency values, the Ra values also increase.

**Figure 3:**
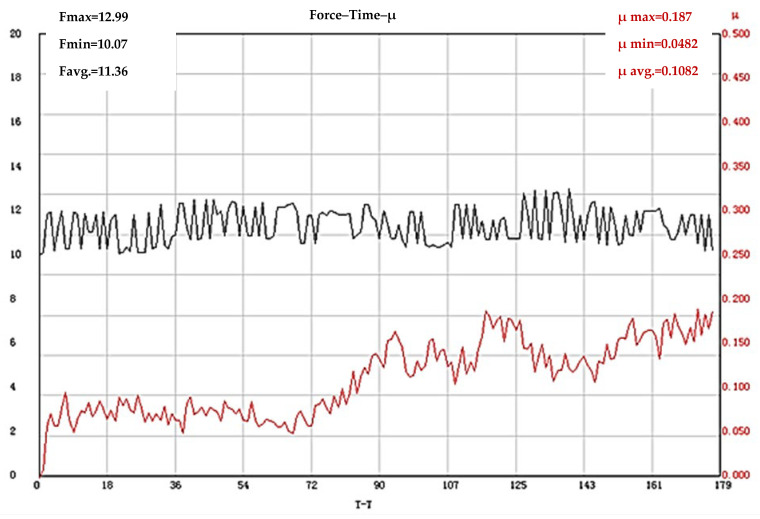
Results of COF determination of X165CrMoV12 steel (not subjected to laser texturing). The black line shows the change in the loading force (F), and the red line shows the coefficient of friction.

The results of tribological tests of test bodies made of hardened and tempered steel X165CrMoV12 (not subjected to laser texturing) show that the stage of tribological system operation is ≈72 s, and this stage is characterized by relatively low COF values. After this stage, the friction coefficient gradually increases and, from ≈90 s to the end of the test, maintains almost the same values with slight fluctuations. The lowest measured value during the test is µ = 0.0482, the highest is µ = 0.187, and the average value of the friction coefficient is µ = 0.1082. It is noteworthy that during the test, the applied force maintains values higher than the set ones, the most likely reason for this being the obtained hardness of the steel after quenching and tempering of 61 HRC, as a result of which the resistance to deformation of the material is high, as well as the increased contact surface between the elements of the tribosystem (Figure 3).

**Figure 4:**
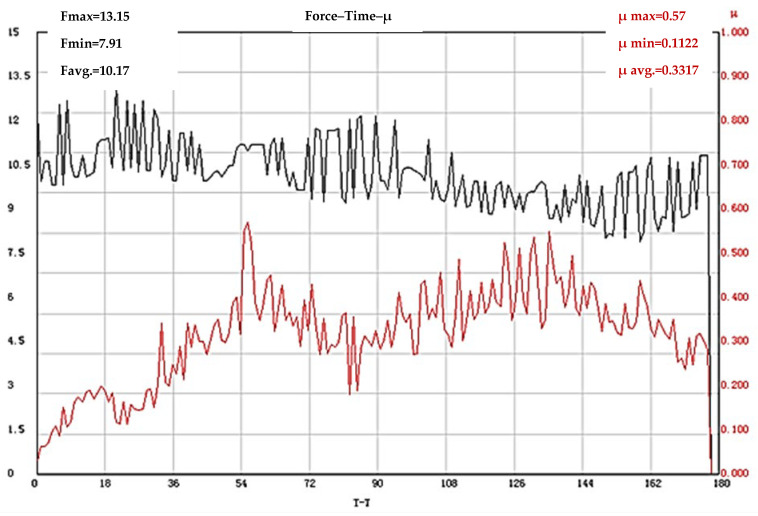
Results of COF determination of X165CrMoV12 steel subjected to quenching and tempering and subsequent laser texturing with an operating frequency of 50 kHz. The black line shows the change in the loading force (F), and the red line shows the coefficient of friction.

The results obtained from the tribological tests of test bodies, which were first subjected to quenching and tempering, after which laser modification was carried out, show that in all three texturing modes used, the friction coefficient is higher compared to that of X165CrMoV12 steel (not subjected to laser processing). For test bodies textured with an operating frequency of 50 kHz, the stage of activation of the tribological system is ≈54 s, and during this stage a constant increase in COF is observed. The lowest measured value in the test process is µ = 0.1122, the highest is µ = 0.57, and the average value of the friction coefficient is µ = 0.3317 (Figure 4).

**Figure 5:**
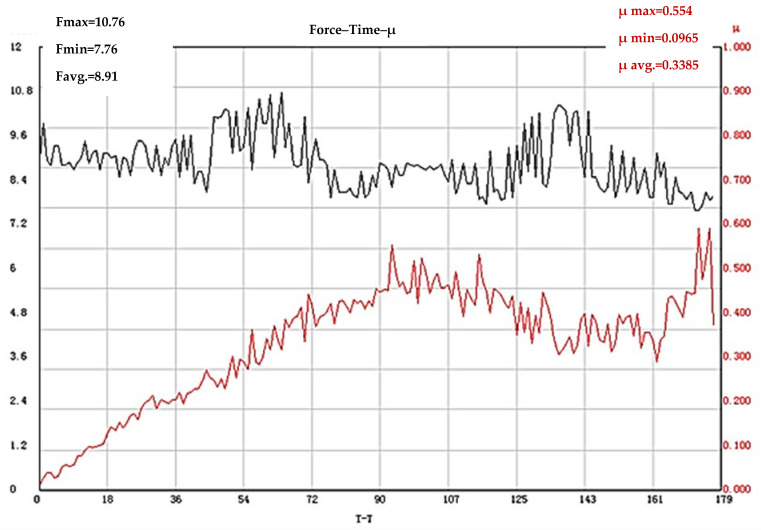
Results of COF determination of X165CrMoV12 steel subjected to quenching and tempering and subsequent laser texturing with an operating frequency of 100 kHz. The black line shows the change in the loading force (F), and the red line shows the coefficient of friction.

The increased working frequency of texturing (100 kHz) leads to increased roughness of the treated surfaces, as a result of which the stage of the tribological system’s running-in is longer at ≈90 s with a constant increase in COF, after which a smooth decrease in the values of the friction coefficient is observed. The greater roughness of the surfaces not only affects the behavior of the tribological pair in the running-in process, but also leads to an increase in the average values of the friction coefficient (µ = 0.3385), which are slightly higher compared to those in the previous texturing mode. The graph obtained in the process of testing test bodies subjected to quenching and tempering and subsequent laser modification with an operating frequency of 100 kHz is shown in Figure 5.

**Figure 6:**
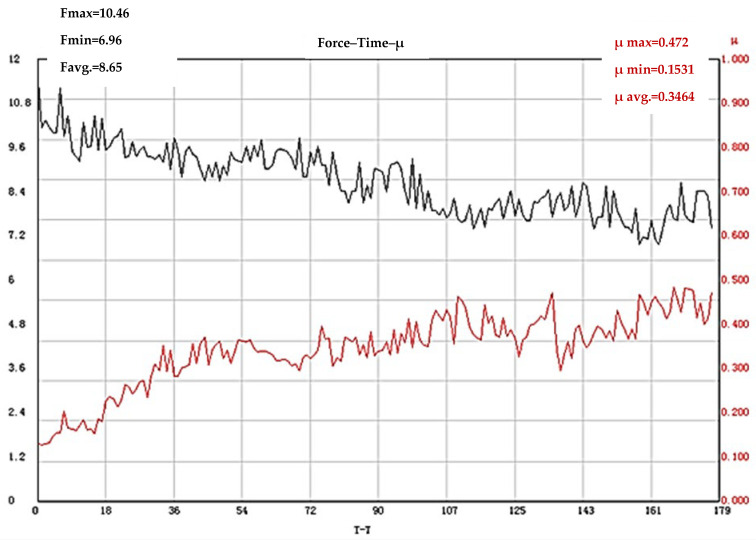
Results of COF determination of X165CrMoV12 steel subjected to quenching and tempering and subsequent laser texturing with an operating frequency of 150 kHz. The black line shows the change in the loading force (F), and the red line shows the coefficient of friction.

Increasing the operating frequency of laser texturing to 150 kHz leads to the registration of the highest values of the coefficient of friction (µ = 0.3464) for the test bodies first subjected to quenching and tempering, and then subjected to laser surface treatment. It has been established that with laser modification with a frequency of 150 kHz, the roughness of the treated surfaces is the greatest, which in turn leads to the longest stage of working-in of the cases considered so far (≈110 s). After this stage, a slight decrease in COF is observed, after which its values increase again until the end of the test (Figure 6).

**Figure 7:**
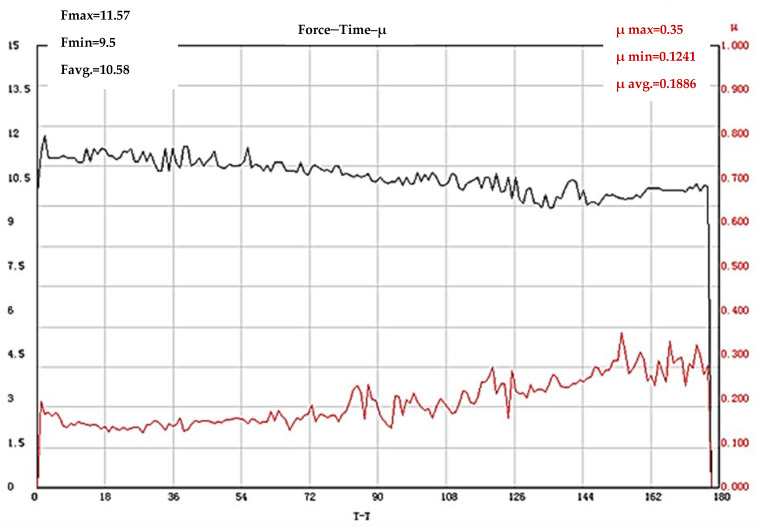
Results of COF determination of X165CrMoV12 steel subjected to laser texturing with an operating frequency of 150 kHz and subsequent quenching and tempering. The black line shows the change in the loading force (F), and the red line shows the coefficient of friction.

The results of the tribological tests conducted to determine the coefficient of friction of test bodies that were first subjected to laser texturing and then hardened and tempered show that in this sequence of processes, the measured COF values are significantly lower compared to those of test bodies that were first subjected to heat treatment and then to laser treatment. It has been established that the operating frequency also has an influence on the friction coefficient, which directly affects the surface roughness. The highest value of the friction coefficient (µ = 0.1886) is registered for test bodies in which a frequency of 150 kHz was used (Figure 7). This COF value is lower than the values obtained in the tests of test bodies that were first subjected to heat treatment and then to laser texturing, regardless of the values of the processing frequency.

**Figure 8:**
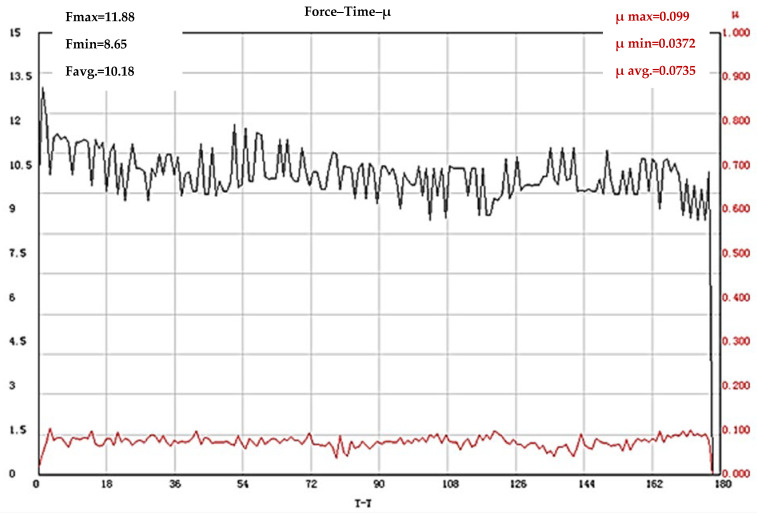
Results of COF determination of X165CrMoV12 steel subjected to laser texturing with an operating frequency of 100 kHz and subsequent quenching and tempering. The black line shows the change in the loading force (F), and the red line shows the coefficient of friction.

**Figure 9:**
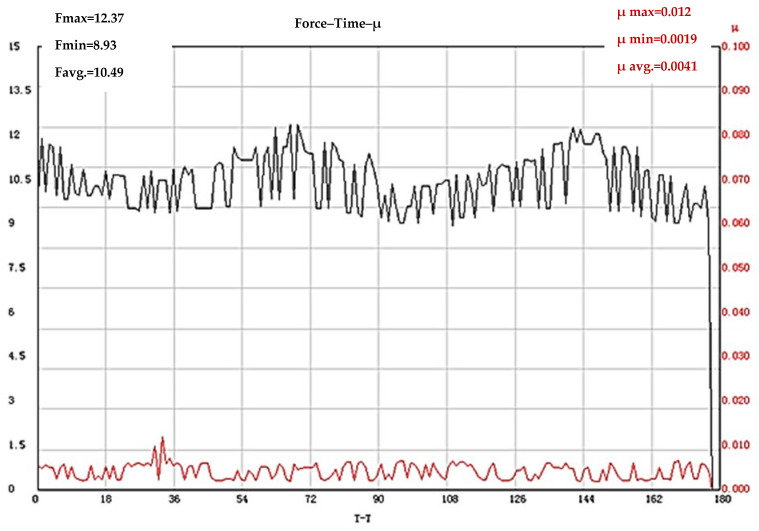
Results of COF determination of X165CrMoV12 steel subjected to laser texturing with an operating frequency of 50 kHz and subsequent quenching and tempering. The black line shows the change in the loading force (F), and the red line shows the coefficient of friction.

The results obtained for COF of the test bodies with the processing frequency of 100 kHz and 50 kHz are of interest. The test bodies that were first subjected to laser texturing with a frequency of 100 kHz and then subjected to heat treatment have a friction coefficient value (µ = 0.0735) much lower than those previously established with a different sequence of processes, but also one significantly lower than the friction coefficient values (µ = 0.1082) of test bodies made of the base hardened X165CrMoV12 steel. The results of the tests of the test bodies that were first subjected to laser processing with a frequency of 100 kHz and then subjected to heat treatment are shown in Figure 8. It was found that the lowest values of the coefficient of friction of all the tribological tests conducted are possessed by the test bodies that were first subjected to laser texturing with an operating frequency of 50 kHz, and then subjected to heat treatment according to the cycle presented in Figure 1. The registered value of the coefficient of friction is µ = 0.0041 (Figure 9).

**Figure 10:**
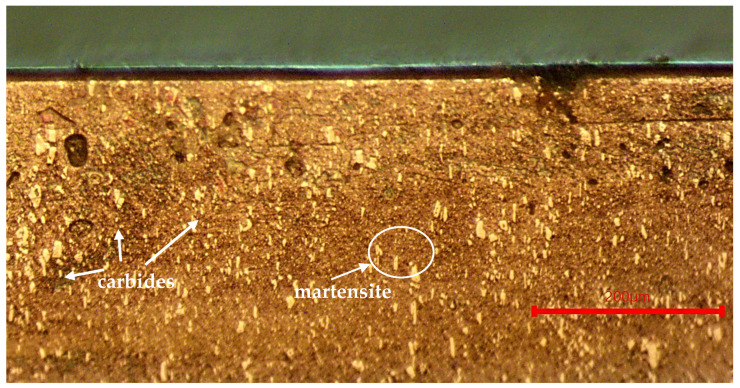
Structure of X165CrMoV12 steel subjected to hardening and triple tempering.

From the diagrams presented in Figure 8 and Figure 9, it is evident that in both tests conducted, no significant changes in the values of the friction coefficient are observed for the entire period of the test. It was established that the use of three different operating frequencies of laser texturing (50 kHz, 100 kHz and 150 kHz) leads to the production of reliefs with different roughness values, which inevitably affects the tribological behavior of the test bodies; however, the significant differences in the friction coefficient under the same modification modes are a prerequisite for studying the influence of the sequence of the processes. By conducting optical metallography of the cross-sections of the test bodies, the resulting structures are studied. The structure of test specimens made of X165CrMoV12 steel subjected to heat treatment according to the scheme presented in Figure 1 consists of martensite and carbides and no decarburization of the surface layer is observed; the specimens have a hardness of 61 HRC (Figure 10).

**Figure 11:**
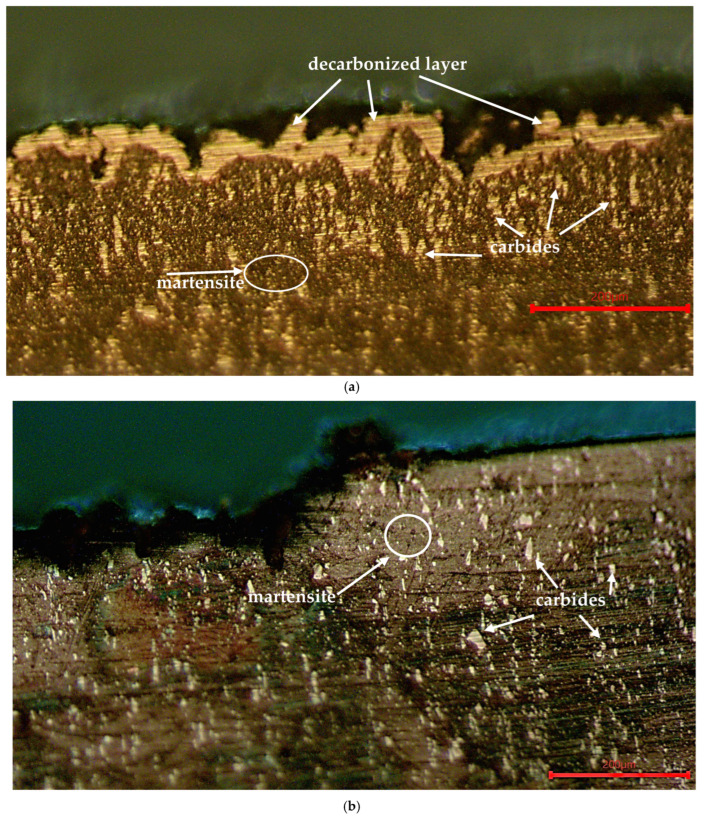
Structure of X165CrMoV12 steel after different sequences of processes used: (**a**) subjected to quenching and tempering and subsequent laser texturing; (**b**) subjected to laser texturing and subsequent quenching and tempering.

The structures obtained with different sequences of laser texturing and heat treatment are of interest. In the test bodies that were first quenched and tempered and then laser-treated (regardless of the operating frequency used), a clearly pronounced decarbonized layer is observed on the textured surface (Figure 11a). No structural changes in depth are recorded, which confirms the notion that the use of laser texturing does not lead to volumetric changes in the structure of the processed material. In the test bodies that were first subjected to laser texturing and then quenched and tempered, in addition to no structural changes in the volume of the test bodies, no defects from carbon loss are recorded on the laser-textured surface (Figure 11b). Invariably, a decarbonized layer is obtained in the laser processing process; however, heating for hardening at the selected working temperature and subsequent holding are the basis of ongoing diffusion processes, in which the amount of carbon in the entire volume of the test bodies is equalized.

The results obtained from the conducted optical metallography show that planning the sequence of the laser texturing and heat treatment processes is of utmost importance for improving the performance properties of the studied X165CrMoV12 steel.

## 4. Discussion

The laser texturing process of test specimens made of X165CrMoV12 steel leads to an increase in the roughness of the treated surfaces, and with increasing operating frequency, the roughness values Ra also increase. The higher values of the roughness of the surfaces of the test specimens subjected first to quenching and tempering and subsequent laser modification lead to higher values of the friction coefficient compared to the base hardened and tempered 61 HRC and Ra 0.607 µm steel. Another reason for the higher COF values obtained in this sequence of processes used is most likely due to a change in the chemical composition and structure of the surface layer (decarburization) as a result of laser processing. This assumption is due to the results obtained from the optical metallography of the test bodies (Figure 11a), which clearly shows a light layer on the textured surface. The reasons for the occurrence of a defect of this nature are most likely due to the lack of use of a protective environment during texturing and the high temperature of the process. At the high temperature of laser ablation, the carbon from the surface layer combines with oxygen from the surrounding atmosphere and forms carbon-containing gases (CO and CO_2_). Chromium is a volatile element that not only evaporates, but also easily forms oxides. The loss of carbon and chromium in the metal matrix prevents the formation of hard carbides of alloying elements (Cr, V, and Mo), which are the basis of the increased hardness of steel. The hardness of martensite directly depends on the carbon dissolved in α-Fe, i.e., the loss of carbon is the reason for the structure’s lower martensite content; thus, the structure is softer and more ductile. From all this, it can be assumed that these zones will have reduced hardness, as a result of which their resistance to plastic deformation will be lower. The resulting softer and more plastic surface is the basis for a more severe tribological process characterized by plastic deformation and wear of the contact surface layer, as a result of which higher values of the friction coefficient compared to the base steel are recorded.

Of greater interest is the behavior in the tribological tests of the test specimens of the studied steel, which were first subjected to laser texturing and then processed according to the used thermal cycle. Although these test specimens have identical roughness to the previously considered samples, their friction coefficient values are significantly lower. It has been established that the test specimens that are laser-modified with an operating frequency of 150 kHz have a friction coefficient value of µ = 0.1886, which is significantly lower than the COF values of the test specimens subjected first to thermal treatment and then to laser texturing, regardless of the values of the processing frequency. In this case, this shows that the resulting structure obtained as a result of the sequence of surface treatment and thermal treatment plays a major role in its tribological behavior. The value of the coefficient of friction of µ = 0.1886 for the test bodies that were laser-modified with an operating frequency of 150 kHz and underwent subsequent heat treatment is higher than the value of the coefficient of friction of µ = 0.1082 for the basic hardened and tempered steel. These data show that in this case, the influence of surface roughness is prioritized, because the test bodies have the same structure in nature.

The coefficient of friction of the test bodies, which were first subjected to laser texturing with an operating frequency of 100 kHz and then heat treated, has a value of µ = 0.0735, which is significantly lower than the COF values of all cases considered so far, including the base hardened X165CrMoV12 steel. From the diagram shown in Figure 8, it is evident that the values of the coefficient of friction do not change significantly throughout the entire testing period. Fluctuations in the COF values around a statistically stable average value are a determining indicator of a dominant wear mechanism, without the occurrence of plastic deformation of the contact surface. This is most likely due to the structure and high hardness of the textured layer, and due to the significantly high roughness, it can be assumed that shearing of the roughness of the surface layer occurs; thus, the bulk material remains protected, and the generation of waste particles is limited—a combination characteristic of stable sliding of the elements of the tribosystem. These considerations are also supported by the change in the values of the applied load during the testing process. An increase in the values of the applied normal load (F) is registered at the beginning of the testing process, after which a decrease is observed and by the end of the tribological process, no significant changes in the force F are registered. In this case, the resulting structure of the textured surface obtained as a result of the choice of the sequence of application of the two processes used, as well as the resulting surface relief, have a positive influence on the friction coefficient, despite the large values of the measured roughness.

It was found that the lowest value of the coefficient of friction (µ = 0.0041) of all the conducted tribological tests is possessed by the test bodies that were first subjected to laser texturing with an operating frequency of 50 kHz, and then subjected to heat treatment according to the used hardening and triple annealing cycle. The COF diagram (Figure 9) shows that the values of the coefficient of friction do not change significantly throughout the entire test period, which in turn is a sign of the occurrence of a tribological process with a dominant wear mechanism, without the occurrence of plastic deformation of the contact surface—a process of stable sliding of the elements of the tribosystem (as in the previous case). In contrast to the graph showing the change in the loading force during the test process of the test bodies that were first subjected to laser texturing with an operating frequency of 100 kHz and then heat treated, in the considered case, two increases in the force (F) to maximum values are established. The test specimens that were laser-modified with an operating frequency of 50 kHz have lower values of surface roughness compared to the test specimens textured with a frequency of 100 kHz. This gives a reason to assume that the time taken for shearing the tips of the contacting finer texture is less, which in turn leads to an increase in the contact surface between the elements of the system and an increase in the loading force (in contrast to the test specimens processed with a frequency of 100 kHz). The second recorded peak with an increase in the loading force to maximum values shows the repeatability of this supposed process of wear of the test specimen texture.

As a result of the heat treatment carried out under dry friction conditions in a tribological pair of steel/steel, the studied tool X165CrMoV12 steel has an average value of the friction coefficient of µ = 0.1082, a value significantly lower than the data presented in the reference literature (0.65–0.80). This shows that the use of three-fold tempering after quenching is relevant, due to it separating a larger amount of secondary carbides and minimizing the amount of residual austenite in the steel structure, i.e., there is a positive effect on the wear resistance of the studied material. When carrying out the processes in the sequence of heat treatment and subsequent laser ablation, higher average values of COF are registered in the three texturing modes (µ = 0.3317, µ = 0.3385, and µ = 0.3464), but they are again lower than the values available in the literature. The expectations that laser texturing will lead to a decrease in the friction coefficient of X165CrMoV12 steel are present in the following sequence of processes: laser texturing and subsequent heat treatment. In this case, a decrease in COF values is observed, such as in Mode 1—50 kHz/pulse energy 0.9 mJ—and Mode 2—100 kHz/pulse energy 0.45 mJ—i.e., they are similar to those indicated in the literature [51,52].

The studied X165CrMoV12 steel is mainly intended for the manufacture of tools operating in conditions of severe abrasive wear and where it is of utmost importance to have high hardness and wear resistance (tools for cold plastic deformation: punching and drawing dies, precision dies, gauges, cutting tools, thread rolling tools, etc.). In practice, during operation, these tools form tribological systems with the blanks and semi-finished products, and therefore the quality of the working surfaces is critical for the quality of the manufactured parts. Laser texturing is a suitable treatment for the working surfaces of the tools, because the formed micro-indentations reduce friction on the contact area, prevent seizing and, when using lubricants, retain friction. Also, the resulting microtexture retains the products separated from wear (working as an abrasive), which in turn facilitates the tribological process. Therefore, laser texturing is a suitable technology for improving the operational properties of the tools and improving the quality of the products.

The conducted experiments show the influence of the operating frequency of laser texturing on the roughness of the resulting surfaces, and the results of the conducted tribological studies are the basis for optimal planning of the laser modification and heat treatment (quenching and tempering) processes of the high-carbon, high-chromium martensitic tool X165CrMoV12 steel.

## 5. Conclusions

- The results of the optical metallography show that when carrying out the laser texturing process of X165CrMoV12 steel in the absence of inert gas, the presence of a decarburized layer in the textured surface is registered.
- The results of the conducted studies show that increasing the operating frequency of the laser modification leads to obtaining surfaces with greater roughness (Mode 1: 50 kHz/pulse energy 0.9 mJ and Ra 4.123 µm; Mode 2: 100 kHz/pulse energy 0.45 mJ and Ra 10.228 µm; Mode 3: 150 kHz/pulse energy 0.3 mJ and Ra 12.489 µm).
- The increase in the operating frequency of laser texturing is the basis for increasing the roughness of the treated surfaces, which in turn affects the values of the friction coefficient. In the sequence of technological processes of thermal treatment–laser ablation, the differences are insignificant due to the presence of a decarburized layer in all three modes. In the sequence of processes of laser modification–thermal treatment, the differences in the COF values are significant.
- The obtained values of the friction coefficient are the lowest in the following sequence of processes: laser texturing and subsequent thermal treatment. The lowest friction coefficient (µ = 0.0041) is registered in the test bodies processed with a mode in which the operating frequency was 50 kHz and the pulse energy was 0.9 mJ, after which they were subjected to thermal treatment according to the used cycle.
- The sequence of the laser texturing and thermal treatment processes, as well as the modification frequency, in which the tool X165CrMoV12 steel has the lowest friction coefficient, has been optimized.

## Figures and Tables

**Table 1:** Chemical composition of steel X165CrMoV12 [wt%].

C	Mn	Si	Cr	Mo	V	Others
1.55–1.75	0.20–040	0.20–0.40	11.00–12.00	0.50–0.70	0.10–0.50	-

**Table 2:** Roughness of laser-textured surfaces at frequencies of 50 kHz, 100 kHz, and 150 kHz.

Roughness	Mode 1—50 kHz/Pulse Energy 0.9 mJ	Mode 2—100 kHz/Pulse Energy 0.45 mJ	Mode 3—150 kHz/Pulse Energy 0.3 mJ
Ra [µm]	4.123	10.228	12.489

## Data Availability

The original contributions presented in this study are included in the article. Further inquiries can be directed to the corresponding authors.
